# Plasma cytokine profiles in HIV-1 infected patients developing neuropathic symptoms shortly after commencing antiretroviral therapy: a case-control study

**DOI:** 10.1186/1471-2334-14-71

**Published:** 2014-02-10

**Authors:** Johan J Van der Watt, Katalin A Wilkinson, Robert J Wilkinson, Jeannine M Heckmann

**Affiliations:** 1 Division of Neurology, Department of Medicine, University of Cape Town, Observatory, Cape Town 7925, South Africa; 2 Department of Medicine, University of Cape Town, Observatory, Cape Town 7925, South Africa; 3 Clinical Infectious Diseases Research Initiative, Institute of Infectious Diseases and Molecular Medicine, University of Cape Town, Observatory, Cape Town 7925, South Africa; 4 Division of Mycobacterial Research, MRC National Institute for Medical Research, London NW7 1AA, UK; 5 Division of Medicine, Imperial College London, London W2 1PG, UK

**Keywords:** HIV neuropathy, Sensory neuropathy, Neuropathic symptoms, Antiretroviral toxicity, Cytokines, Immune reconstitution, Interleukin-1 receptor antagonist

## Abstract

**Background:**

In patients infected with human immunodeficiency virus 1 (HIV-1) neuropathic symptoms may develop within weeks of starting combination antiretroviral therapy (cART). This timing coincides with the occurrence of immune reconstitution inflammatory syndrome. Our objective was to investigate the longitudinal association of plasma cytokine and soluble receptor concentrations with incident neuropathic symptoms within 12 weeks of starting programme-based cART in a nested case-control study.

**Methods:**

One hundred and twenty adults without neuropathic symptoms and about to initiate cART were followed longitudinally for 24 weeks after cART initiation. Subjects were examined for peripheral neuropathy at baseline (pre-cART) and 2-, 4-, 12- and 24 weeks thereafter. Individuals developing neuropathic symptoms within 12 weeks of starting cART were matched in a nested case-control design with those remaining symptom-free for at least 24 weeks. Plasma was collected at each visit. Cytokines and soluble receptors were quantified using multiplex immunometric assays.

**Results:**

Incident neuropathic symptoms occurred in 32 (27%) individuals within 12 weeks of starting cART for the first time. Cytokine concentrations increased at 2 weeks, irrespective of symptom-status, returning to baseline concentrations at 12 weeks. Compared to the control group, the symptomatic group had higher baseline levels of interleukin-1 receptor (IL-1R)-antagonist. The symptomatic group also showed greater increases in soluble interleukin-2 receptor-alpha and tumour necrosis factor (TNF) receptor-II levels at week 2 and soluble interleukin-6 receptor levels at week 12. Ratios of pro-inflammatory- vs anti-inflammatory cytokines were higher for TNF-alpha/IL-4 (p = 0.022) and interferon-gamma/IL-10 (p = 0.044) in those developing symptoms. After 24 weeks of cART, the symptomatic group showed higher CD4+ counts (p = 0.002).

**Conclusions:**

The initiation of cART in previously treatment naïve individuals was associated with a cytokine 'burst’ between 2- and 4 weeks compared with pre-cART levels. Individuals developing neuropathic symptoms within 12 weeks of starting cART showed evidence of altered cytokine concentrations even prior to initiating cART, most notably higher circulating IL-1R-antagonist levels, and altered ratios of “pain-associated” cytokine and soluble receptors shortly after cART initiation.

## Background

Distal sensory polyneuropathy (DSP) is the most prevalent neuropathy in individuals infected with human immunodeficiency virus 1 (HIV-1) and occurs in up to 57% [1,2]. HIV-1-associated DSP includes two clinically identical neuropathies arising either as a consequence of HIV-1 infection (HIV-DSP) or a treatment-induced toxicity from combination antiretroviral therapy (cART) predominantly as a result from exposure to the di-deoxynucleoside reverse transcriptase inhibitors (dNRTI’s). HIV-1 associated DSP is characterized by a high frequency of positive sensory neuropathic symptoms such as pain and paresthesiae [1]. The development of incident neuropathic symptoms peaks within 12 weeks of starting cART [3].

The pathogenesis of HIV-1-associated DSP remains obscure, although likely many factors are at play [4]. For HIV-DSP, the most widely accepted pathogenic mechanism involves neuronal damage secondary to immune activation with release of cytokines and oxygen-radicals [5]. Mitochondrial dysfunction is associated with exposure to nucleoside reverse-transcriptase inhibitor (NRTI) therapy [6] and believed to contribute to the pathogenesis of sensory neuropathy after cART initiation [7]. Despite well-documented *in vitro* studies on mitochondrial DNA polymerase-γ inhibition by NRTIs, studies have not consistently demonstrated a correlation between mitochondrial dysfunction and the presence of NRTI-related adverse effects [8]. Not all individuals treated with NRTIs develop adverse effects related to mitochondrial toxicity. Host factors such as differences in the intracellular phosphorylation of NRTIs, drug–drug or drug–nutrient interactions, and nadir CD4+ counts have been suggested to influence susceptibility towards mitochondrial dysfunction [9-12].

Substantial evidence implicates inflammation and cytokines in the development of neuropathic pain. In animal models, peripheral nerve injury leads to rapid and sustained changes in cytokine expression [13,14]. The intraneural application of pro-inflammatory cytokines induces pain-associated behavioural signs [15] which diminish when treated with anti-inflammatory cytokines [16]. In HIV-1-negative individuals with painful- compared to painless neuropathies, higher blood mRNA levels of tumour necrosis factor-alpha (TNFα) and interleukin (IL)-2 have been reported [17].

Few studies have assessed the role of cytokines in HIV-1-associated DSP. In a cross-sectional cohort, largely on cART, several markers of immune activation in the plasma and cerebrospinal fluid (CSF) were studied; only CSF macrophage colony-stimulating factor levels predicted time to developing symptomatic DSP [18]. A study of cytokine gene polymorphisms in individuals with DSP after cART initiation, with or without symptoms, showed an association with the *TNFA*-locus [19]. Increased TNFα and reduced IL-4 mRNA levels were also found in peripheral nerve tissue from AIDS patients with DSP [20]. Individuals with HIV-1-associated DSP compared to those without were found to have persistently higher levels of chemokine ligand 5 (CCL5) during the first 48 weeks after cART [21].

Upon starting cART, immune reconstitution is accompanied by a rise in CD4+ count and partial restoration of pathogen-specific immunity [22]. However, 8-43% of patients will experience pathological inflammatory responses that may cause clinical deterioration, termed immune reconstitution inflammatory syndrome (IRIS) [23,24]. Typically, IRIS occurs during the first 12 weeks of cART initiation [25], and in the setting of HIV-1 and tuberculosis co-infection, has been shown to be associated with increased circulating pro-inflammatory cytokines TNFα, IL-6, and interferon-gamma (IFN-γ) [26].

As IRIS manifests within a similar period to the development of incident neuropathic symptoms after cART initiation we hypothesized that an exaggerated cytokine response or immune reconstitution after cART initiation contributes to the immunopathogenesis of neuropathic symptoms [3]. We investigated the association between neuropathic symptoms and the concentrations of markers previously shown to be associated with painful neuropathies and/or immune reconstitution, within 12 weeks after cART initiation. These markers included high-sensitivity C-reactive protein (hs-CRP), CD4+ count as well as specific cytokines TNFα, IL-2, IL-4, IL-6 and their soluble receptors.

## Methods

The University of Cape Town Research Ethics Committee approved the study (REC REF 221/2008). All subjects provided written informed consent.

### Study site, subjects and study design

HIV-1-infected individuals who were treatment naïve, but scheduled to start cART within the next seven days were recruited from Crossroads Community Health Centre (Cape Town, South Africa) between May 2009 and December 2010. Individuals were eligible if ≥18 years and met criteria for cART initiation in the government-sponsored HIV treatment programme, i.e. a CD4+ count ≤350 cells/mm^3^.

Individuals with pre-existing symptoms at the baseline visit, were excluded from further analysis. In addition, those with previous exposure to cART, diabetes mellitus, active opportunistic infection, additional systemic illness or inflammatory conditions, current infection with tuberculosis, severe diarrhoea, co-morbid neurological disease that may confound DSP, exposure to glucocorticoids within preceding six months, or pregnancy, were excluded.

Clinical assessments were performed on all eligible participants at baseline (pre-cART) and 2-, 4-, 12-, and 24 weeks thereafter. Fasting plasma samples were obtained at each visit. Peripheral nervous system examination was performed by one of two trained clinicians using the Brief Peripheral Neuropathy Screen and a modified version of Total Neuropathy Score (TNSr) as previously described [1]. These tools include assessments of neuropathic symptoms of pain, numbness, and paraesthesiae on a visual analogue scale (VAS)(0-10) as well as neurological signs including pinprick and vibratory sensation, reflexes, and strength assessments.

The symptomatic group comprised individuals developing neuropathic symptoms within 12 weeks of starting cART. A nested case-control cohort was selected consisting of 30 individuals who did not develop symptoms for 24 weeks after cART initiation (control group). The groups were paired using risk factors previously reported such as age (difference ≤3 years), gender, previous anti-tuberculous treatment, baseline CD4+ count (difference ≤25 cells/mm^3^) and dNRTI containing regimens [1,27,28].

### Sampling and cytokine assays

Sampling was performed at baseline, 2-, 4- and 12 weeks thereafter. Fasting venous blood was collected between 08h00-09h00 to reduce variability (diurnal variations or food ingestion). Samples were immediately placed on ice until centrifuged (<3 hours) and stored at -80°C.

Plasma concentrations of granulocyte-macrophage colony-stimulating factor (GM-CSF), IFNγ, IL-1β, IL-2, IL-4, IL-5, IL-6, IL-7, IL-8, IL-10, IL-12(p70), IL-13, and TNFα were quantified using a multiplexed (Bio-Plex platform) immunometric assay (MILLIPLEX xMAP High Sensitivity Human Cytokine Panel, Millipore, Billerica, MA, USA). Plasma cytokine receptor concentrations of soluble IL (sIL)-1 receptor I (sIL-1RI), sIL-1 receptor II (sIL-1RII), sIL-2 receptor-α (sIL-2Rα), sIL-4 receptor (sIL-4R), sIL-6 receptor (sIL-6R), sTNF receptor I (sTNFRI), and sTNF receptor II (sTNFRII) were quantified using MILLIPLEX xMAP Human Soluble Cytokine Receptor Panel. IL-l receptor antagonist (IL-1RA) was quantified by using MILLIPLEX xMAP Human Cytokine/Chemokine Immunoassay. Assays were carried out in compliance with the kit manufacturer. Concentrations lower than the kit’s limit of detection were extrapolated from a standard curve. Each subject’s samples were prepared on the same plate to minimize inter-plate variability.

High-sensitivity CRP was measured by a particle-enhanced immunoturbidimetric assay (Roche Diagnostics, Mannheim, Germany). CD4+ counts, week 24 viral load, and white cell counts were obtained from chart reviews.

### Statistical analysis

Stata (version 12.0; Texas, USA) was used. Baseline characteristics, cytokine, and soluble receptor concentrations were summarized by proportions or median with interquartile range (IQR). Normality was assessed numerically and log-transformation was applied when necessary. Baseline differences between groups were assessed by *Chi*-square or Fisher’s exact test and Student-*t* or Mann-Whitney U test, as appropriate. Repeated measures analysis (random effects model with cross-sectional time series regression) assessed longitudinal patterns (over time and between groups). Time since cART was used as the within-individual factor and group (symptomatic vs symptom-free) as the between-individual factor. To exclude the effect of random variation and significant fluctuations within an individual’s cytokine peak measurements over time, a Spearman rank correlation analysis was performed. All p-values were two-sided at 5% significance. P-values were not corrected for multiple comparisons as specific candidate markers were selected in a pre-established hypothesis.

## Results

### Patient characteristics

All baseline examinations were performed a median of one day prior to cART initiation (IQR 1-5 days). One-hundred and eighty-four eligible patients were screened at baseline and 24 with neuropathic symptoms were excluded. A further 40 subjects defaulted either due to pregnancy, migration, withdrawal of consent or developed tuberculosis. For the nested sample, cases and controls were selected from the prevalent cohort (n = 120) without symptoms at the baseline visit and who were followed for 24 weeks. Within the first 12 weeks of starting cART, 32 (27%) of these individuals developed neuropathic symptoms; two were excluded from further analysis because of detectable viral loads at week 24 and the remaining 30 comprised the symptomatic group.

Of the individuals who remained symptom-free for 24 weeks, 30 were selected as controls after matching them with the symptomatic group for known risk factors such that no differences were observed in baseline characteristics (Table 1). The baseline characteristics of the matched controls did not differ from the overall cohort without neuropathic symptoms (n = 88; Table 1). Fasting glucose (median = 4.7; normal 4.1-5.6 mmol/L) and triglycerides levels (median = 0.8; normal 0.5-2.0 mmol/L) were normal and similar between the symptomatic- and control groups (p ≥ 0.53, results not shown) as well as alcohol consumption (any alcohol in the preceding year, 31%; p = 0.83).

**Table 1:** Baseline (pre-cART) clinical and laboratory characteristics by incident neuropathic symptom status

		**Nested case (neuropathic symptoms) vs control (symptom-free) sample**		
**Variable**	**Total cohort remaining symptom-free N = 88**	**Neuropathic symptom group N = 30**	**Control group N = 30**	**Total N = 60**
**Female sex**, N (%)	62 (70)	22 (73)	22 (73)	44 (73)
Age^*^, years	31 (27 – 38)	32 (26 - 35)	34 (26 - 41)	33 (26 - 37)
Weight^*^, kg	63 (56- 72)	58.0 (52.5 - 68.5)	59.5 (54.0 - 64.0)	58.5 (53.0 - 68.0)
Height^*^, m	1.61 (1.56 - 1.69)	1.58 (1.55 - 1.64)	1.62 (1.57 - 1.65)	1.61 (1.55 - 1.64)
Body Mass Index^*^, kg/m^2^	23.3 (20.3 - 28.0)	22.7 (20.1 - 28.0)	22.6 (19.8 - 25.3)	22.6 (19.8 - 28.0)
**dNRTI**^†^, N (%)	68 (77)	24 (40)	22 (37)	46 (77)
*D4T/3TC/NNRTI*	68 (77)	24 (40)	22 (37)	46 (77)
*AZT/3TC/NNRTI*	5 (6)	2 (7)	3 (10)	5 (8)
*TDF/3TC/NNRTI*	15 (17)	4 (13)	5 (17)	9 (15)
**Previous TB**, N (%)	25 (28)	7 (23)	7 (23)	14 (23)
*< 1 year ago*	13 (15)	3 (10)	3 (10)	6 (10)
*1 year ago*	1 (1)	0 (0)	0 (0)	0 (0)
*2 years ago*	2 (2)	0 (0)	1 (3)	1 (2)
*> 2 years ago*	9 (10)	4 (13)	3 (10)	7 (12)
Years since HIV diagnosis^*^	1 (1-3.4)	1 (1-3.5)	2 (1-4)	1 (1-3.8)
**WHO stage**, N (%)				
*Stage 1*	30 (34)	6 (20)	12 (40)	18 (30)
*Stage 2*	25 (28)	18 (60)	10 (33)	28 (47)
*Stage 3*	30 (34)	5 (17)	7 (23)	12 (20)
*Stage 4*	3 (3)	1 (3)	1 (3)	2 (3)
White cell count^*^, ×10^9^/L	5.1 (4.0 - 6.6)	5.7 (3.9 - 6.4)	5.7 (4.4 - 7.9)	5.7 (4.3 - 7.1)
CD4 T-cell count^*^, cells/mm^3^	163 (125 - 193)	145 (109 - 181)	138 (110 - 194)	141 (110 - 189)
**CD4 T-cell count**, N (%)				
*< 100 cells/mm*^*3*^	15 (17)	5 (17)	6 (20)	11 (18)
*100 - 200 cells/mm*^*3*^	56 (64)	19 (63)	20 (67)	39 (65)
*> 200 cells/mm*^*3*^	17 (19)	6 (20)	4 (13)	10 (17)
hs-CRP^*^, mg/L	2.6 (0.9 - 6.5)	2.6 (0.9 - 5.4)	2.9 (0.9 - 6.7)	2.7 (0.9 - 6.7)

The total cohort remaining symptom-free refers to individuals who were asymptomtic at baseline and remained free of neuropathic symptoms in the first 6 months of starting cART; the nested control group were extracted from this total cohort and paired (with the neuropathic symptom group) for previously identified risk factors. The neuropathic symptom group refers to individuals developing symptoms within 12 weeks of starting cART.

*Abbreviations*: *dNRTI* dideoxy-nucleoside reverse-transcriptase inhibitor (d-drug), *D4T* stavudine, *3TC* lamivudine, *AZT* zidovudine, *TDF* tenofovir, *TB* tuberculosis, *WHO* World Health Organization, *hs-CRP* high-sensitivity C-reactive protein.

^*^Median (Interquartile range).

^†^dNRTI refers to d-drug containing combination antiretroviral therapy regimen commenced subsequent to baseline assessment. NNRTI refers to non-nucleoside reverse transcriptase inhibitor and were either efavirenz or nevirapine.

The median age of the nested case-control group was 33 years (range 20-47). More than half of the individuals had been diagnosed with HIV-1 infection within a year preceding the baseline visit; overall, there was no difference between the case- and control groups (p = 0.22). The WHO HIV stage, baseline white cell count (median = 5.7 × 10^9^/l), CD4+ count (median = 141/mm^3^) and hs-CRP (median = 2.7 mg/L) were similar, as were weight and height measurements. Baseline HIV-1 viral loads were unavailable.

### Characteristics of subjects developing neuropathic symptoms

The peak incidence in symptoms was between weeks 4 and 12 (14%) (Table 2). Overall, estimated crude incidence rate at 24 weeks was 30%. The most frequent symptom was paraesthesiae (25/30), followed by numbness (21/30) and pain (15/30). Numbness never occurred as an isolated neuropathic symptom. At initial presentation the median pain/paresthesiae severity by VAS was 4 (IQR 1-6).

**Table 2:** Incidence of neuropathic symptoms within 24 weeks after cART initiation

**Interval (weeks)**	**Incident SN**	**r**_**1**_	**R**_**1**_
**0 – 2**	6	0.06	0.06
**2 – 4**	10	0.10	0.15
**4 – 12**	16	0.14	0.27
**12 – 24**	4	0.04	0.30

r_1_ is calculated as the ratio of the number of individuals who developed neuropathic symptoms to the number of patients at risk during the interval.

R_1_ is the cumulative rate of development of neuropathic symptoms by end of the interval.

For further analysis, 2 individuals with incident neuropathic symptoms were excluded due to detectable HIV-1 viral loads at week 24.

Eleven of 30 individuals developing incident symptoms had distal neuropathic signs at baseline; six had hypoactive ankle reflexes compared to the knees, four had reduced pin sensibility and one reduced vibration perception. Incident symptoms were accompanied by new/worsening neuropathic sign(s) in 15 (50%). By week 24 after cART initiation, pain or paresthesiae had resolved in 17 (transient symptoms), whereas the remainder had persistent or worsening symptoms.

### Plasma cytokines in both groups

**Figure 1:**
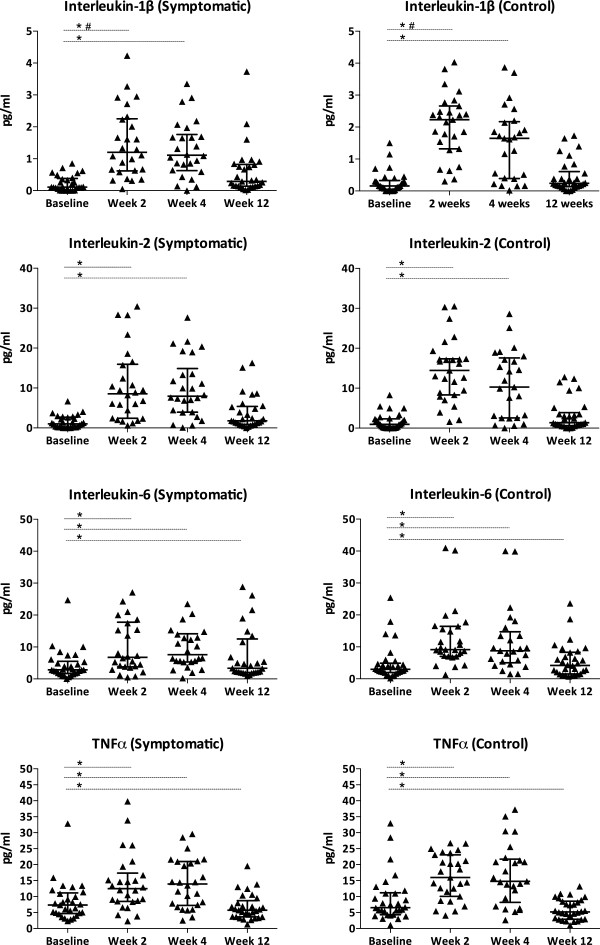
**Longitudinal evaluation of candidate cytokine concentrations categorized by incident neuropathic symptom status.** Incident neuropathic symptoms developed within 12 weeks of starting cART. The control group refers to the symptom-free nested control group paired for previously identified risk factors. *refers to within-group differences from baseline, p < 0.05. # refers to between-group differences from baseline levels, p < 0.05. Horizontal bars indicate median values with interquartile ranges. Baseline refers to pre-cART levels. Abbreviations: IL, interleukin; TNFα, tumour necrosis factor-α.

Baseline pre-cART cytokine concentrations were similar between the symptomatic and control groups (Figure 1 and Additional file 1: Table S1). Median concentrations of IL-4, IL-12(p70) and IL-13 were below the minimum detectable level at baseline (Additional file 1: Table S2).

The initiation of cART significantly affected both pro- and anti-inflammatory cytokine concentrations, irrespective of the development of neuropathic symptoms. Almost all the cytokines measured demonstrated significantly higher concentrations at week 2 and week 4 returning to baseline levels by week 12, and included IL-1β, IL-2, IL-4, IL-5, IL-6, IL-7, IL-10, IL-12p(70), IL-13, IFNγ, GM-CSF and TNFα (Figure 1 and Additional file 1: Table S1). The exception was IL-8 with lower levels at week 12 compared to baseline. Irrespective of symptom status, TNFα increased from pre-cART levels, peaked between weeks 2 and 4, but then decreased at week 12 to a lower level than at baseline (p = 0.008). At week 12, IL-6 levels remained higher compared to that at baseline (p = 0.033). Cytokine levels showed within-individual stability and predictability by correlating significantly at two different time points for 11 cytokines, and at three time points for four of the 11 (Additional file 1: Table S3).

The pro-inflammatory T-helper (Th)-1 cytokines showed the greatest increases from baseline concentrations; IL-12p(70) (≈700-fold), IL-2 (≈300-fold) and IFNγ (≈200-fold). TNFα concentrations increased 3-fold. Compared with baseline, Th2 cytokine levels also responded to cART initiation; at week 2, IL-13 increased 59-fold and IL-4 36-fold. IL-5 and IL-10 increased by comparatively modest amounts (4- to 5-fold). The hs-CRP levels increased in both groups compared to pre-cART levels, and peaked at week 2 (p = 0.001) without significant differences by symptom status (Table 3).

**Table 3:** CD4+ count and high-sensitivity CRP pre-cART and longitudinally over 12 weeks, categorized by neuropathic symptom status

**Variable**	**Time point**	**Time effect**	**Group effect**	
		**Median (IQR), pg/ml**	**Neuropathic symptom group Median (IQR)**	**Control group Median (IQR)**
**CD4+ count**, cells/mm^3^	*Baseline*	141 (110; 189)	145 (109; 181)	138 (110; 194)
	*Week 24*	**272 (204; 368)**	**298 (210; 457)**	**260 (179; 351)**
**hs-CRP**, mg/L	*Baseline*	2.70 (0.90; 6.70)	2.90 (0.90; 6.70)	2.60 (0.90; 5.40)
	*Week 2*	**6.70 (2.10; 18.50)**	8.40 (3.40; 17.00)	5.30 (1.80; 19.30)
	*Week 4*	6.50 (3.30; 26.50)	8.40 (3.50; 30.60)	4.20 (3.30; 15.00)
	*Week 12*	3.95 (1.50; 7.20)	3.80 (1.50; 8.30)	4.20 (1.00; 6.70)

Incident neuropathic symptoms developed within 12 weeks of starting cART.

The control group refers to the symptom-free nested control group paired for previously identified risk factors.

Time effect: Bold numbers denote values different from baseline, p < 0.05.

Group effect: Bold numbers denote values different between groups compared to baseline, p < 0.05.

*Abbreviations*: IQR interquartile range, *hs-CRP* high-sensitivity C-reactive protein.

### Plasma cytokines in the symptomatic group compared to the control group

Despite the general increase in cytokine concentrations, there was a tendency for lower peak concentrations in the symptomatic group compared to the control group. However, individuals in the symptomatic group with persistent symptoms throughout the 24-week period compared to those developing transient symptoms, showed a significantly greater IL-6 peak at week 2 (p = 0.023) (Additional file 1: Table S4). Symptom severity, categorized by a VAS cut-off of 4/10, did not associate with cytokine concentrations at any sampling point (Additional file 1: Table S4). After 24 weeks of cART, the symptomatic group showed greater CD4+ count reconstitution compared with those remaining symptom-free (298 cells/mm^3^; IQR 210-457 vs 260 cells/mm^3^; IQR 179-351, p = 0.002) (Table 3).

Stratifying the overall cohort by dNRTI exposure or CD4+ counts >100 cells/mm^3^ did not show an association with cytokine concentrations at any sampling point (Results not shown).

### Soluble cytokine receptors

The half-life of soluble cytokine receptors is longer than that of cytokines. Therefore, soluble cytokine receptors may be more informative of chronic inflammation [29]. In the symptomatic group compared to the control group, the baseline median IL-1RA concentrations were significantly higher (10.2 vs 1.9 pg/mL, p = 0.03), and remained higher throughout the study period (Table 4). However, 26 of 60 individuals (6 symptomatic and 20 symptom-free) had baseline IL-1RA concentrations lower than the detectable level.

**Table 4:** Soluble receptor and IL-1RA concentrations pre-cART and longitudinally over 12 weeks, categorized by neuropathic symptom status

**Cytokine**	**Time point**	**Time effect**	**Group effect**	
		**Median (IQR) (pg/mL)**	**Neuropathic symptom-group Median (IQR) (pg/mL)**	**Control group Median (IQR) (pg/mL)**
**sIL-1RI**	*Baseline*	29.6 (23.1; 41.0)	33.3 (23.2; 43.1)	25.5 (23.0; 34.3)
	*Week 2*	34.0 (26.6; 45.6)	36.9 (29.0; 48.3)	33.6 (25.2; 39.5)
	*Week 4*	37.6 (28.2; 45.6)	37.5 (27.4; 46.1)	38.7 (28.9; 42.9)
	*Week 12*	28.2 (23.4; 38.9)	34.5 (26.3; 44.2)	25.2 (22.5; 35.0)
**sIL-1RII**	*Baseline*	4572 (1904.5; 6339.5)	4160.6 (1593.2; 5550.0)	4922.8 (2017.9; 6489.5)
	*Week 2*	4885.7 (2144.1; 7056.3)	4744.4 (2144.1; 6174.4)	4905.8 (2027.0; 8988.9)
	*Week 4*	5116.7 (2453.1; 7658.8)	5115.7 (2453.1; 7443.9)	5751.6 (2674.1; 8659.4)
	*Week 12*	5806.5 (2726.7; 8628.2)	5604.6 (2622.7; 8295.6)	6152.1 (2845.9; 8651.8)
**sIL-2rα**	*Baseline*	1102.3 (728.1; 2021.5)	1068.2 (699.0; 2005.3)	1210.3 (757.1; 2383.1)
	*Week 2*	1049.2 (595.1; 1781.7)	**1188.4 (638.5; 1831.3)**	**866.1 (472.3; 1589.2)**
	*Week 4*	**881.2 (580.8; 1763.4)**	745.5 (595.0; 1230.0)	1032.1 (558.8; 1763.4)
	*Week 12*	**733.3 (434.6; 1073.1)**	647.1 (432.9; 869.5)	823.8 (610.9; 1264.5)
**sIL-6R**	*Baseline*	9379.3 (6441.9; 12912.4)	9379.3 (6386.6; 12726.3)	9545.9 (6518.4; 15032.9)
	*Week 2*	9480.6 (7325.3; 14352.6)	10038.4 (7421.2; 14014.9)	9183.6 (6446.9; 15126.8)
	*Week 4*	11538.0 (8288.9; 14148.6)	11248.9 (7994.0; 12857.9)	11940.5 (9510.9; 14148.6)
	*Week 12*	**8123.9 (6241.2; 12582.7)**	**8250.4 (6978.9; 12582.7)**	**7518.7 (5366.6; 11001.6)**
**sTNFRI**	*Baseline*	536.2 (288.7; 845.4)	551.4 (292.6; 860.6)	489.4 (284.7; 749.7)
	*Week 2*	560.9 (347.2; 799.5)	647.5 (417.4; 803.7)	516.4 (302.3; 782.2)
	*Week 4*	606.9 (379.0; 872.3)	646.0 (424.7; 742.0)	571.0 (379.0; 913.7)
	*Week 12*	455.1 (347.2; 750.5)	532.9 (416.5; 753.8)	415.2 (319.4; 612.3)
**sTNFRII**	*Baseline*	5441.7 (4425.5; 7722.1)	5441.7 (4285.5; 8470.5)	5582.4 (4539.0; 7230.2)
	*Week 2*	**5104.3 (3728.7; 6909.2)**	**5680.6 (4255.6; 7181.9)**	**4236.9 (3629.6; 6032.3)**
	*Week 4*	**4757.1 (3718.4; 6621.7)**	4307.4 (3531.6; 6486.4)	5766.0 (4121.2; 6922.4)
	*Week 12*	**4564.9 (3532.0; 6127.8)**	4485.4 (3113.5; 6384.8)	4579.4 (3924.5; 5791.2)
**IL-1RA**	*Baseline*	4.4 (0.0; 16.7)	**10.2 (2.1; 18.8)**	**1.9 (0.0; 8.0)**
	*Week 2*	3.4 (0.0; 16.7)	11.3 (0.8; 25.8)	0.4 (0.0; 14.0)
	*Week 4*	5.4 (0.0; 13.1)	5.7 (0.0; 11.5)	2.1 (0.0; 14.0)
	*Week 12*	3.4 (0.0; 14.0)	5.1 (0.8; 15.1)	0.0 (0.0; 10.2)

Incident neuropathic symptoms developed within 12 weeks of starting cART.

The control group refers to the symptom-free nested control group paired for previously identified risk factors.

Time effect: Bold numbers denote values different from baseline, p < 0.05.

Group effect: Bold numbers denote values different between groups compared to baseline, p < 0.05.

*Abbreviations*: *IQR* interquartile range, *sIL-1RI* soluble IL-1 receptor I, *sIL-1RII* soluble IL-1 receptor II, *sIL-2Rα* soluble IL-2 receptor-α, *sIL-4R* soluble IL-4 receptor, *sIL-6R* soluble IL-6 receptor, *sTNFRI* soluble TNF receptor I, *sTNFRII* soluble TNF receptor II, *IL-1RA* IL-l receptor antagonist.

After starting cART, concentrations of sTNFRII, sIL-2Rα and sIL-6R all declined significantly in both groups from baseline levels over the 12-week study period, albeit at different sampling points (Table 4); sTNFRII was significantly lower at 2 weeks, sIL-2Rα at 4 weeks, and sIL-6R at 12 weeks. However, a repeated measures analysis showed that concentrations of sIL-2Rα and sTNFRII two weeks after cART initiation, were significantly higher in the group developing neuropathic symptoms compared to the control group (sTNFRII, p = 0.031; sIL-2Rα, p = 0.034)(Table 4). Soluble receptors sIL-1RI, sIL-1RII and sTNFRI remained relatively stable throughout.

### Pro-inflammatory/anti-inflammatory cytokine ratios and cytokine/soluble receptor ratios

**Figure 2:**
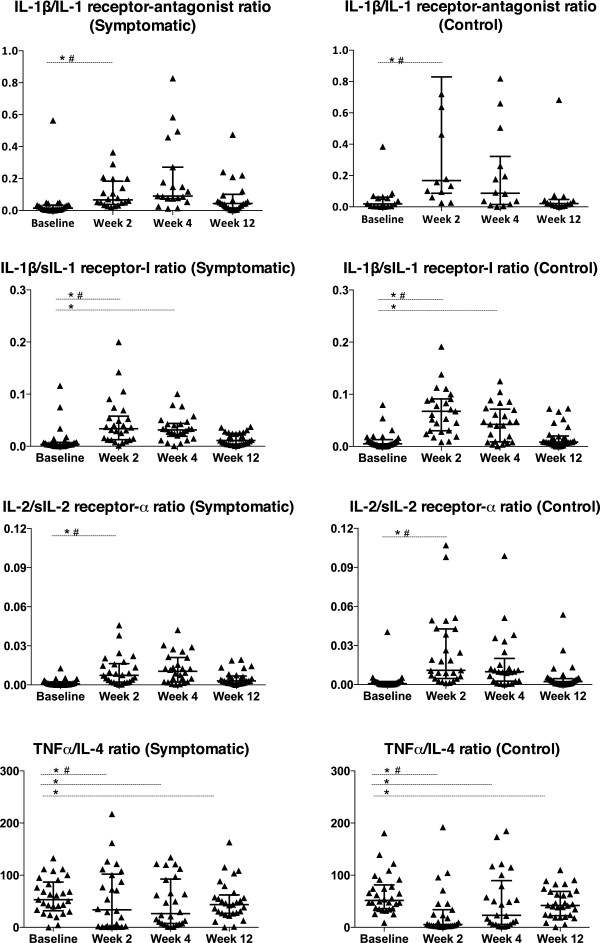
**Longitudinal evaluation of candidate pro-inflammatory/anti-inflammatory cytokine and cytokine/receptor ratios categorized by incident neuropathic symptom status.** Incident neuropathic symptoms developed within 12 weeks of starting cART. The control group refers to the symptom-free nested control group paired for previously identified risk factors. *refers to within-group differences from baseline, p < 0.05. # refers to between-group differences from baseline levels, p < 0.05. Horizontal bars indicate median values with interquartile ranges. Baseline refers to pre-cART levels. Abbreviations: IL, interleukin; sIL, soluble interleukin; TNFα, tumour necrosis factor-α.

The balance of pro- and anti-inflammatory cytokines may be as important as individual cytokine concentrations. Therefore, we assessed ratios of pro- and anti-inflammatory cytokines and their soluble receptors as markers of a dysregulated immune response [29-32]. At week 2, the symptomatic group compared with the control group showed lower ratios of cytokines to soluble cytokine receptors: IL-1β/sIL-1RI (p = 0.004), IL-1β/IL-1RA (p = 0.006) and IL-2/sIL-2Rα (p = 0.014) (Figure 2). At the same time point, the “pain-associated” cytokines of *a priori* interest showed higher ratios in the symptomatic group for TNFα/IL-4 (p = 0.022) and a trend for IL-6/IL-4 (p = 0.054) and IFNγ/IL-4 (p = 0.061). At week 12 the symptomatic group still had a higher IFNγ/IL-10 ratio (p = 0.044) (Figure 2).

## Discussion

The development of incident neuropathic symptoms within 12 weeks of starting cART developed in 27% of our community-based cohort. The peak period of developing neuropathic symptoms was between 2 and 12 weeks, with 10% occurring in 2-4 weeks and 14% between 4-12 weeks. Few prior studies have evaluated neuropathy status at 12 weeks, all with incomplete data [3,33]; at 24 weeks, our crude incidence rate of 30% is similar to that of a Ugandan cohort reporting 38% with incident neuropathic symptoms [33].

Comparison of plasma cytokines in the symptomatic and control groups showed significant differences in only two cytokines at 2 weeks (IL-1β and IL-13, Additional file 1: Table S1). However, baseline IL-1RA were 5-times higher in the symptomatic group preceding the clinical onset of symptoms and the initiation of cART. IL-1RA has features of an acute phase protein [29] suggesting that in individuals susceptible to developing neuropathic symptoms, there may be a higher set-point of sub-clinical inflammation pre-cART. Two weeks after starting cART, the sIL-2Rα and sTNFRII also showed higher peak concentrations in the symptomatic group compared with the controls remaining symptom-free. Furthermore, at 24 weeks the symptomatic group showed greater reconstitution of the CD4+ cell count.

Not all subjects developing symptoms remained symptomatic by 24 weeks. Those with persistent symptoms at 24 weeks showed higher circulating IL-6 levels when compared to those with transient neuropathic symptoms. Importantly, two individuals with detectable viral loads at 24 weeks were excluded to minimize the confounding effect of uncontrolled HIV-1 infection. Taken together these results suggest that patients developing symptoms within the first 12 weeks after starting cART experience enhanced sub-clinical inflammation and immune reconstitution in the systemic compartment compared to those remaining symptom-free.

Previously, higher levels of systemic TNFα- and IL-2-mRNA were reported in HIV-1-negative painful neuropathies when compared with painless neuropathies [17]. Although in our study plasma IL-2 and TNFα concentrations did not mirror the soluble receptor levels or segregate by symptom status, the systemic cytokine concentrations of both were substantially lower than the concentrations of their respective soluble receptors (sIL-2Rα, sTNFRI and sTNFRII). A possible explanation is that IL-2 and TNFα, secreted by Th1 cells, act locally in an autocrine or a paracrine manner and are neutralized systemically by their soluble receptors as an immune regulatory mechanism [34]. Furthermore, the longer plasma half-life of these soluble receptors and their rapid biological inactivation of systemic cytokines suggest that the elevated levels detected in some soluble receptors in the symptomatic group may indicate a relatively higher level of inflammation [35,36].

In order to further explore markers of immune dysregulation we assessed ratios of key cytokines previously implicated in neuropathic pain [17,20]. The ratios of TNFα/IL-4 and IL-6/IL-4 were significantly higher at 2 weeks and IFNγ/IL-10 at 12 weeks in the symptomatic group compared to the control group. These findings suggest that in those developing symptoms, there is an imbalance between pro- and anti-inflammatory cytokines in which Th1 subsets and their cytokine products (IL-6, IFNγ and TNFα), dominate over immunoregulatory Th2 subsets and their cytokines ( IL-4 and IL-10).

A novel observation was the transient but significant burst in both pro- and anti-inflammatory plasma cytokine concentrations, as well as hs-CRP levels, within 2-4 weeks after starting cART in both groups irrespective of symptom status. Twelve of the 13 cytokines analysed increased significantly within 4 weeks of cART initiation. Cytokines such as IL-2, IL-12(p70) and IFNγ increased by more than 200-times relative to pre-cART/baseline concentrations before returning to baseline levels at 12 weeks. Although Padilla et al. also found increased levels of IL-6 and hs-CRP at four weeks compared to baseline, their cohort comprised a mixture of subjects either initiating cART or re-starting [37].

Although peak cytokine concentrations in this cohort overall were lower than those reported in hospitalized tuberculosis-associated IRIS patients [26,38], this study population comprised ambulatory individuals attending a community-based clinic. Parasitic- and non-parasitic infections, as well as nutritional deficiencies could contribute to the degree of observed immune activation once the HIV-induced functional inhibition is reversed by cART.

We speculate that the trend for lower cytokine peaks in the symptomatic group may be the consequence of systemic neutralization by the concomitant and significantly higher concentration of IL-1RA and soluble receptors. For example, IL-1RA is released from hepatocytes stimulated by circulating inflammatory mediators, and also synthesized by locally activated monocytes [39,40]. Circulating IL-1RA during inflammatory stress may serve to reduce the systemic responses to localized IL-1β production. High levels of circulating IL-1RA have been reported in many inflammatory settings including target-organ autoimmune diseases such as rheumatoid arthritis [29]. The pre-cART IL-1β/IL-1RA ratio in our control group was similar to a previous and comparable cohort of asymptomatic African women with reasonably preserved CD4+ counts who were also cART-naive [41]. By comparison, the group developing neuropathic symptoms showed a markedly altered IL-1β/IL-1RA ratio pre-cART as a result of significantly higher IL-1RA levels. We propose that higher plasma IL-1RA levels in those who subsequently develop symptoms reflect the host’s homeostatic mechanism aimed at downregulating pre-existing inflammation. This may result in nerve dysfunction, possibly at the dorsal root ganglion (DRG) when the blood-nerve barrier is impaired [41], causing neuropathic symptoms after an additional insult such as dNRTIs or the observed 'cytokine burst’ after cART intitiation. Although the factors driving the higher inflammatory set-point in individuals predicted to develop early incident neuropathic symptoms is unknown, careful attention to micro-nutritional status and concomitant drug usage prior to starting cART may provide simple measures to reduce cellular oxidative stress known to augment inflammation.

The DRG plays a pivotal role in the pathogenesis of HIV-associated neuropathy which likely involves both indirect cytokine- and direct viral protein-mediated neurotoxicity [42]. Neuronal toxicity in the DRG is associated with upregulated IL-1β and TNFα expression [42,43]. Subclinical levels of inflammation such as local IL-1β production, may prime proteinase-activated receptors (PAR)_2_ on small DRG neurons and their afferent axons [44]. A “second hit” such as the cART-associated subclinical “cytokine burst” herein described, may facilitate further PAR_2_ activation with augmentation of nociceptive signaling via central pathways [44,45].

Our study has several limitations. Plasma levels may not reliably reflect perineural activation due to the local action of cytokines at low concentrations. Therefore, systemic cytokine levels might not accurately demonstrate the extent of neural inflammation in individuals developing neuropathic symptoms. In addition, cytokines and soluble receptor levels showed significant skewing and variability. Fluctuation in cytokine levels may have affected results although rank correlation analyses demonstrated good within-individual stability and predictability for the majority of cytokines. A random effects model was used to limit the effect of variability and fluctuations on the longitudinal analysis of cytokine levels. Although the two individuals with detectable viral loads at 24 weeks were excluded from the analysis, a degree of undetectable viral replication may have confounded the interpretation of the cytokine results. A further limitation is that CD4 counts were not available at the 12 week analysis point to correlate with systemic cytokine levels. Also, our conclusions are based on a small, matched sample, reducing our statistical power to detect weaker associations. This prevalent cohort of HIV-infected individuals were “selected” at the baseline visit for being free of neuropathic symptoms prior to cART exposure, which in itself may have introduced selection bias. The symptomatic cases and symptom-free controls were matched for factors known to increase the risk for DSP, but there may be other factors, as yet unknown, that may also influence risk. Finally, individuals with diabetes mellitus and current opportunistic infections including tuberculosis (and anti-tuberculous therapy) were excluded from our study and we therefore do not know how these factors might have influenced our results.

## Conclusion

In this case-control study of asymptomatic subjects starting cART we show significant increases in cytokine levels within the first 2-4 weeks after initiating therapy, a time when susceptible individuals typically manifest pathological IRIS. We found that individuals developing neuropathic symptoms within 12 weeks of cART initiation have higher baseline levels of IL-1RA even prior to starting cART. Our conjecture is that this is evidence of a chronic host response to inflammatory stimuli prior to cART in those individuals who develop neuropathic symptoms. An additional “insult” such as initiating cART with the observed cytokine burst and higher ratios of pain-associated cytokines (TNFα/IL-4, IL-6/IL-4 and IFNγ/IL-10), and concomitant increase in soluble cytokine receptors (sIL-2Rα and sTNFRII), might give rise to neuropathic symptoms via complex direct and indirect signalling mechanisms.

## Competing interests

The authors declare that they have no competing interests.

## Authors’ contributions

JJvdW collected, assembled and analyzed the data. JJvdW, KAW, RJW, and JMH contributed to the study design, interpretation of data, writing and revision of the article. All authors read and approved the final manuscript.

## Pre-publication history

The pre-publication history for this paper can be accessed here:

http://www.biomedcentral.com/1471-2334/14/71/prepub

## Supplementary Material

Additional file 1 **Supplementary Tables. Table S1**– Cytokine concentrations before cART initiation and longitudinally over the first 12 weeks categorized by incident neuropathic symptom status. **Table S2** – The baseline cytokine concentrations, minimum detectable concentrations, as well as the percentage of undetectable levels for each cytokine. **Table S3** – Spearman rank correlation coefficients between baseline and all follow-up visits. **Table S4** – Longitudinal effect of symptom duration and severity of symptoms on candidate cytokine levels. Click here for file
