# Supplementary material for: Plasma cytokine profiles in HIV-1 infected patients developing neuropathic symptoms shortly after commencing antiretroviral therapy: a case-control study
Source: BMC Infect Dis. 2014 Feb 10;14:71. doi: 10.1186/1471-2334-14-71 (PMC3928502; doi:10.1186/1471-2334-14-71)
Supplement: Additional file 1 — Supplementary Tables. Table S1– Cytokine concentrations before cART initiation and longitudinally over the first 12 weeks categorized by incident neuropathic symptom status. Table S2 – The baseline cytokine concentrations, minimum detectable concentrations, as well as the percentage of undetectable levels for each cytokine. Table S3 – Spearman rank correlation coefficients between baseline and all follow-up visits. Table S4 – Longitudinal effect of symptom duration and severity of symptoms on candidate cytokine levels. [file 1471-2334-14-71-S1.docx]

# Additional file 1

# Supplementary tables

## Table S1 – Cytokine concentrations before cART initiation and longitudinally over the first 12 weeks categorized by incident neuropathic symptom status.

| **Cytokine** | **Time point** | **Time effect** | **Group effect** | |
| --- | --- | --- | --- | --- |
|  |  | **Median (IQR)**  **(pg/mL)** | **Neuropathic symptom group Median (IQR)**  **(pg/mL)** | **Control group Median (IQR)**  **(pg/mL)** |
| **IL-1**β | *Baseline* | 0.12 (0.01 ; 0.36) | 0.11 (0.01 ; 0.36) | 0.16 (0.03 ; 0.30) |
|  | *Week 2* | **1.71 (0.75 ; 2.46)** | **1.20 (0.63 ; 2.23)** | **2.23 (1.32 ; 2.66)** |
|  | *Week 4* | **1.31 (0.51 ; 1.97)** | 1.11 (0.64 ; 1.69) | 1.65 (0.40 ; 2.14) |
|  | *Week 12* | 0.26 (0.14 ; 0.77) | 0.29 (0.14 ; 0.81) | 0.24 (0.14 ; 0.55) |
| **IL-2** | *Baseline* | 0.99 (0.14 ; 2.38) | 1.01 (0.18 ; 2.63) | 0.95 (0.08 ; 2.27) |
|  | *Week 2* | **10.73 (6.01 ; 17.20)** | 8.54 (2.42 ; 15.76) | 14.44 (8.33 ; 17.35) |
|  | *Week 4* | **9.90 (3.08 ; 17.16)** | 7.94 (3.96 ; 13.50) | 10.29 (2.57 ; 17.16) |
|  | *Week 12* | 1.69 (0.80 ; 5.07) | 1.80 (0.90 ; 5.26) | 1.34 (0.67 ; 3.49) |
| **IL-4** | *Baseline* | 0.12 (0.12 ; 0.12) | 0.12 (0.12 ; 0.12) | 0.12 (0.12 ; 0.12) |
|  | *Week 2* | **1.21 (0.12 ; 7.20)** | 0.12 (0.12 ; 8.36) | 2.79 (0.12 ; 7.06) |
|  | *Week 4* | 0.12 (0.12 ; 2.69) | 0.13 (0.12 ; 2.73) | 0.12 (0.12 ; 2.50) |
|  | *Week 12* | 0.12 (0.12 ; 0.12) | 0.12 (0.12 ; 0.12) | 0.12 (0.12 ; 0.12) |
| **IL-5** | *Baseline* | 0.56 (0.32 ; 1.11) | 0.54 (0.30 ; 1.23) | 0.58 (0.32 ; 1.10) |
|  | *Week 2* | **1.94 (1.20 ; 3.29)** | 1.28 (0.84 ; 3.48) | 2.30 (1.71 ; 3.29) |
|  | *Week 4* | **1.83 (1.09 ; 2.85)** | 2.01 (1.07 ; 2.42) | 1.83 (1.17 ; 3.18) |
|  | *Week 12* | 0.77 (0.43 ; 1.29) | 0.76 (0.49 ; 1.04) | 0.85 (0.39 ; 1.34) |
| **IL-6** | *Baseline* | 2.84 (1.92 ; 5.11) | 2.84 (1.77 ; 5.32) | 2.97 (1.99 ; 4.66) |
|  | *Week 2* | **8.71 (4.50 ; 16.43)** | 6.79 (3.86 ; 17.46) | 9.15 (7.01 ; 16.43) |
|  | *Week 4* | **8.71 (5.31 ; 13.94)** | 7.62 (5.31 ; 13.94) | 8.79 (5.42 ; 13.46) |
|  | *Week 12* | **4.09 (2.01 ; 8.62)** | 3.37 (2.07 ; 11.55) | 4.21 (1.53 ; 8.23) |
| **IL-7** | *Baseline* | 2.06 (0.67 ; 3.68) | 2.04 (0.59 ; 4.49) | 2.06 (0.87 ; 3.57) |
|  | *Week 2* | **9.02 (4.45 ; 15.41)** | 7.45 (3.57 ; 16.12) | 11.58 (5.91 ; 15.41) |
|  | *Week 4* | **7.56 (3.28 ; 13.12)** | 7.00 (3.86 ; 10.28) | 9.18 (3.28 ; 13.12) |
|  | *Week 12* | 2.66 (1.27 ; 5.64) | 2.81 (1.32 ; 5.64) | 2.48 (1.27 ; 3.74) |
| **IL-8** | *Baseline* | 3.77 (2.46 ; 5.25) | 3.38 (2.43 ; 4.81) | 4.30 (2.49 ; 5.52) |
|  | *Week 2* | 3.28 (2.30 ; 4.98) | 2.94 (2.04 ; 4.74) | 3.86 (2.38 ; 6.05) |
|  | *Week 4* | 3.21 (2.32 ; 4.77) | 3.13 (2.34 ; 4.36) | 3.21 (2.32 ; 4.88) |
|  | *Week 12* | **3.52 (2.21 ; 4.24)** | 3.16 (2.21 ; 4.66) | 3.68 (1.86 ; 4.15) |
| **IL-10** | *Baseline* | 14.91 (7.03 ; 29.35) | 14.31 (7.24 ; 28.31) | 16.25 (6.97 ; 36.05) |
|  | *Week 2* | **36.28 (24.37 ; 55.30)** | 36.68 (18.84 ; 55.30) | 35.68 (25.43 ; 55.79) |
|  | *Week 4* | **31.82 (16.91 ; 53.23)** | 30.94 (20.69 ; 48.84) | 33.99 (12.83 ; 55.06) |
|  | *Week 12* | 10.68 (5.01 ; 25.60) | 13.99 (6.18 ; 24.52) | 9.89 (4.79 ; 28.16) |
| **IL-12p(70)** | *Baseline* | 0.01 (0.01 ; 1.02) | 0.01 (0.01 ; 0.95) | 0.02 (0.01 ; 1.08) |
|  | *Week 2* | **13.69 (6.10 ; 22.83)** | 8.55 (1.53 ; 20.76) | 17.84 (8.22 ; 23.04) |
|  | *Week 4* | **9.46 (3.44 ; 19.55)** | 8.42 (3.63 ; 17.63) | 12.53 (3.44 ; 19.55) |
|  | *Week 12* | 1.11 (0.01 ; 3.86) | 1.11 (0.01 ; 4.51) | 1.29 (0.01 ; 2.92) |
| **IL-13** | *Baseline* | 0.47 (0.47 ; 7.26) | 0.47 (0.47 ; 9.80) | 0.47 (0.47 ; 7.21) |
|  | *Week 2* | **37.20 (17.25 ; 72.83)** | **25.39 (12.49 ; 51.92)** | **57.21 (21.06 ; 76.60)** |
|  | *Week 4* | **32.10 (11.75 ; 49.28)** | 30.04 (17.93 ; 44.73) | 33.28 (11.30 ; 51.79) |
|  | *Week 12* | 3.12 (0.47 ; 16.63) | 3.12 (0.47 ; 16.63) | 2.83 (0.47 ; 13.20) |
| **IFNγ** | *Baseline* | 1.20 (0.01 ; 3.23) | 1.05 (0.01 ; 3.08) | 1.38 (0.01 ; 3.77) |
|  | *Week 2* | **12.53 (6.16 ; 20.92)** | 8.42 (3.79 ; 25.01) | 12.82 (11.32 ; 20.05) |
|  | *Week 4* | **8.86 (3.12 ; 18.01)** | 9.52 (3.32 ; 18.13) | 8.86 (2.87 ; 15.09) |
|  | *Week 12* | 2.41 (0.39 ; 6.93) | 3.32 (1.34 ; 6.94) | 1.54 (0.01 ; 4.85) |
| **GM-CSF** | *Baseline* | 0.73 (0.17 ; 1.51) | 0.76 (0.18 ; 1.42) | 0.71 (0.15 ; 1.63) |
|  | *Week 2* | **6.41 (2.99 ; 10.08)** | 4.62 (2.28 ; 7.95) | 8.37 (4.78 ; 11.04) |
|  | *Week 4* | **3.39 (1.44 ; 8.55)** | 3.10 (2.27 ; 6.11) | 3.92 (1.44 ; 9.47) |
|  | *Week 12* | 1.04 (0.57 ; 2.72) | 1.17 (0.57 ; 2.72) | 0.96 (0.57 ; 2.53) |
| **TNFα** | *Baseline* | 6.64 (4.53 ; 10.98) | 7.36 (4.84 ; 10.90) | 6.53 (4.35 ; 10.98) |
|  | *Week 2* | **14.19 (8.68 ; 20.68)** | 12.47 (8.51 ; 16.69) | 15.98 (10.05 ; 23.05) |
|  | *Week 4* | **14.52 (7.38 ; 21.12)** | 13.94 (7.38 ; 20.94) | 14.76 (9.49 ; 21.30) |
|  | *Week 12* | **5.52 (3.33 ; 8.44)** | 5.71 (3.64 ; 7.96) | 5.20 (2.92 ; 8.44) |

Incident neuropathic symptoms developed within 12 weeks of starting cART.

The control group refers to the nested control group paired for previously identified risk factors.

Time effect: Bold numbers denote values different from baseline values, p<0.05.

Group effect: Bold numbers denote values different between groups compared to baseline levels, p<0.05.

Abbreviations: IQR, interquartile range; IL, interleukin; GM-CSF, granulocyte-macrophage colony-stimulating factor; IFNγ, interferon-gamma; TNFα, tumour necrosis factor-alpha.

## Table S2 – The baseline cytokine concentrations, minimum detectable concentrations, as well as the percentage of undetectable levels for each cytokine.

| **Cytokine** | **Baseline median (IQR) (pg/mL)** | **Minimum detectable concentration (pg/mL)** | **Undetected N(%)** |
| --- | --- | --- | --- |
| **IL-1β** | 0.12 (0.01 ; 0.36) | 0.06 | 17 (29%) |
| **IL-2** | 0.99 (0.14 ; 2.38) | 0.16 | 16 (27%) |
| **IL-4** | 0.12 (0.12 ; 0.12) | 0.13 | 58 (97%) |
| **IL-5** | 0.56 (0.32 ; 1.11) | 0.01 | 1 (2%) |
| **IL-6** | 2.84 (1.92 ; 5.11) | 0.10 | 1 (2%) |
| **IL-7** | 2.06 (0.67 ; 3.68) | 0.12 | 4 (7%) |
| **IL-8** | 3.77 (2.46 ; 5.25) | 0.11 | 0 (0%) |
| **IL-10** | 14.91 (7.03 ; 29.35) | 0.15 | 0 (0%) |
| **IL-12** | 0.01 (0.01 ; 1.02) | 0.11 | 37 (62%) |
| **IL-13** | 0.47 (0.47 ; 7.26) | 0.48 | 34 (57%) |
| **IFNγ** | 1.20 (0.01 ; 3.23) | 0.29 | 22 (37%) |
| **GM-CSF** | 0.73 (0.17 ; 1.51) | 0.46 | 23 (38%) |
| **TNFα** | 6.64 (4.53 ; 10.98) | 0.05 | 0 (0%) |

Abbreviations: IQR, interquartile range; IL, interleukin; GM-CSF, granulocyte-macrophage colony-stimulating factor; IFNγ, interferon-gamma; TNFα, tumour necrosis factor-alpha.

## Table S3 – Spearman rank correlation coefficients between baseline and all follow-up visits

| **Cytokine** | **Week 2** | **Week 4** | **Week 12** |
| --- | --- | --- | --- |
| **IL-1β** | 0.22 | 0.52^**^ | 0.22 |
| **IL-2** | 0.15 | 0.44^*^ | 0.29 |
| **IL-4** | 0.22 | 0.14 | 0.29 |
| **IL-5** | 0.40^*^ | 0.69^**^ | 0.56^**^ |
| **IL-6** | 0.50^**^ | 0.57^**^ | 0.19 |
| **IL-7** | 0.28 | 0.41^*^ | 0.41^*^ |
| **IL-8** | 0.26 | 0.14 | 0.19 |
| **IL-10** | 0.49^**^ | 0.71^**^ | 0.54^**^ |
| **IL-12** | 0.30 | 0.52^**^ | 0.28 |
| **IL-13** | 0.20 | 0.43^*^ | 0.35 |
| **IFNγ** | 0.43^*^ | 0.68^**^ | 0.39^*^ |
| **GM-CSF** | 0.23 | 0.50^**^ | 0.47^**^ |
| **TNFα** | 0.33 | 0.63^**^ | 0.47^**^ |

^*^ p < 0.05

^**^ p < 0.01

Abbreviations: IL, interleukin; GM-CSF, granulocyte-macrophage colony-stimulating factor; IFNγ, interferon-gamma; TNFα, tumour necrosis factor-alpha.

## Table S4 – Longitudinal effect of symptom duration and severity of symptoms on candidate cytokine levels

| **Cytokine** | **Symptom duration group effect** | | **Symptom severity group effect** | |
| --- | --- | --- | --- | --- |
|  | **Coefficient** | **Confidence interval (95%)** | **Coefficient** | **Confidence interval (95%)** |
| **IL-1β** |  |  |  |  |
| *Week 2* | -0.39 | (-1.31 ; 0.52) | -0.15 | (-0.97 ; 0.68) |
| *Week 4* | 0.02 | (-0.91 ; 0.94) | 0.24 | (-0.58 ; 1.07) |
| *Week 12* | 0.08 | (-0.85 ; 1.01) | 0.28 | (-0.52 ; 1.09) |
| **IL-2** |  |  |  |  |
| *Week 2* | -2.89 | (-9.48 ; 3.71) | -1.97 | (-7.93 ; 4.00) |
| *Week 4* | 1.05 | (-5.60 ; 7.69) | 2.67 | (-3.29 ; 8.64) |
| *Week 12* | -1.51 | (-8.23 ; 5.21) | 0.44 | (-5.35 ; 6.24) |
| **IL-6** |  |  |  |  |
| *Week 2* | **-11.50** | **(-21.43 ; -1.56)** | 7.92 | (-1.37 ; 17.21) |
| *Week 4* | -3.66 | (-13.69 ; 6.37) | 6.61 | (-2.68 ; 15.89) |
| *Week 12* | -7.90 | (-18.05 ; 2.25) | 5.75 | (-3.20 ; 14.70) |
| **TNFα** |  |  |  |  |
| *Week 2* | -3.02 | (-8.81 ; 2.78) | -4.89 | (-10.22 ; 0.44) |
| *Week 4* | -1.18 | (-7.02 ; 4.67) | -1.22 | (-6.55 ; 4.10) |
| *Week 12* | -1.00 | (-6.91 ; 4.91) | -2.04 | (-7.19 ; 3.10) |

Symptom duration group effect: Difference in mean cytokine concentration between individuals with resolved symptoms at 24 weeks compared to those with unresolved symptoms.

Symptom severity group effect: Difference in mean cytokine concentration between symptom grade ≥ 2 severity (VAS≥4) compared to grade 1 severity.

Bold numbers denote values different between groups compared to baseline levels, p<0.05.

Abbreviations: IL, interleukin; TNFα, tumour necrosis factor-alpha.
